# Cognition and mobility show a global association in middle- and late-adulthood: Analyses from the Canadian Longitudinal Study on Aging

**DOI:** 10.1016/j.gaitpost.2018.06.116

**Published:** 2018-07

**Authors:** Naiara Demnitz, David B. Hogan, Helen Dawes, Heidi Johansen-Berg, Klaus P. Ebmeier, Marc J. Poulin, Claire E. Sexton

**Affiliations:** a Department of Psychiatry, University of Oxford, Oxford, United Kingdom; b Wellcome Centre for Integrative Neuroimaging, FMRIB, John Radcliffe Hospital, University of Oxford, United Kingdom; c Division of Geriatric Medicine, Department of Medicine, Cumming School of Medicine, University of Calgary, Canada; d Centre for Movement and Occupational Rehabilitation Sciences, Oxford Institute of Nursing, Midwifery and Allied Health Research, Oxford Brookes University, United Kingdom; e Department of Physiology and Pharmacology and the Hotchkiss Brain Institute, Cumming School of Medicine and Faculty of Kinesiology, University of Calgary, Canada; f Wellcome Centre for Integrative Neuroimaging, Oxford Centre for Human Brain Activity, Department of Psychiatry, University of Oxford, Oxford, United Kingdom; g Global Brain Health Institute, Memory and Aging Center, Department of Neurology, University of California, San Francisco, CA, USA

**Keywords:** Gait, Balance, Physical function, Cognitive aging, CLSA

## Abstract

**Background:**

Given our aging population, there’s great interest in identifying modifiable risk factors for cognitive decline. Studies have highlighted the relationship between aspects of mobility and cognitive processes. However, cognition and mobility are both multifaceted concepts and their interrelationships remain to be well defined.

**Research question:**

Here, we firstly aimed to replicate cross-sectional associations between objective measures of mobility and cognition. Second, we tested whether these associations remained after the consideration of multiple age-related confounders. Finally, to test the hypothesis that the association between mobility and cognition is stronger in older adults, we examined the moderating effect of age in the association between mobility and cognition.

**Methods:**

In the Canadian Longitudinal Study on Aging, 28,808 community-dwelling adults (aged 45–87; 51% female) completed mobility (gait, balance and chair stands) and cognitive (memory, executive function and processing speed) assessments. General linear models were used to examine mobility-cognition relationships and the moderating effect of age.

**Results:**

Cognitive measures were significantly associated with mobility measures (all p < 0.001). Further, age significantly moderated the mobility-cognition relationship, with the strength of the associations generally increasing with age.

**Significance:**

All cognitive measures were related to indices of mobility, suggesting a global association. In our moderation analyses, the mobility-cognition relationship often increased with age. However, the small effect sizes observed suggest that mobility is, in isolation, not a strong correlate of cognitive performance in middle and late-adulthood.

## Highlights

• Examined associations between mobility and cognitive measures in 28,808 adults.
• All cognitive measures were related to mobility, suggesting a global association.
• Associations remained after accounting for multiple age-related confounders.
• The association between mobility and cognition tends to increase with age.

## 1 Introduction

Age-related neural changes are associated with a decline in various cognitive domains, such as memory, executive function, and processing speed. Similarly, age-related neural and muscular changes often lead to a deterioration in aspects of mobility, such as gait, balance and lower-extremity function. These changes may occur in tandem, as mounting evidence has highlighted the relationship between aspects of cognition and mobility processes [1]. Given that cognition and mobility impairments are a costly burden both to the individual and to society, the drive to identify correlates of healthy ageing warrants a better understanding of cognition-mobility relationships.

Cognition and mobility are both multifaceted concepts, and the interrelationship between their sub-domains is not yet well characterised. Whereas some have suggested that mobility is preferentially associated with executive function [2], a recent meta-analysis of cross-sectional studies on cognition and mobility in healthy older adults found evidence for associations across the board, albeit with small effect sizes [3]. Further, studies have often focused on the association between one mobility and one cognitive measure (e.g. gait and executive function), thus limiting conclusions about the global nature of the mobility-cognition association.

The interdependence between mobility and cognition has been hypothesised to reflect age-dependent changes in shared neural mechanisms [4] and an increased demand for cognitive monitoring in motor control with age [5]. However, previous studies have predominantly focused on older adults, resulting in an unclear understanding of the effect of age on this association. Accordingly, examining the mobility-cognition relationship in a wider age-span may lead to a clearer picture of its development.

The present study aimed to conduct the largest investigation of mobility and cognitive performance to date, through analysis of a population-based dataset with a wide age-span, the Canadian Longitudinal Study on Aging (CLSA). In a component of the CLSA study, psychological and mobility baseline data were collected on over 30,000 adults, aged between 45 and 85. Here, we firstly aimed to replicate cross-sectional associations between objective measures of mobility (walking time, chair stands and balance) and cognition in the baseline CLSA data. Since these associations have typically been studied with sample sizes of N < 400, or more recently with samples in the 1000s [6,7], a sample size in the 10,000s may serve as a powerful response to the discrepancies that remain in the mobility-cognition literature. Enabled by our large sample size, we also tested whether these associations remained after the consideration of multiple age-related confounders. Finally, to test the hypothesis that the association between mobility and cognition is stronger in older adults, we examined the moderating effect of age in the association between mobility and cognition.

## 2 Methods

### 2.1 Participants

The CLSA is a Canadian multi-centre study of 51,338 people between the ages of 45 and 85 years at the time of recruitment [8]. All CLSA participants provided data on demographic, lifestyle, physical, clinical, psychological and economic measures. A sub-set of CLSA participants (n = 30,097), referred to as the CLSA Comprehensive cohort, visited a local data collection site for additional assessments, including physical performance measures and additional cognitive testing. For the present analysis, only participants from the CLSA Comprehensive cohort (dataset version 2.1) were considered. Participants missing all cognitive or all mobility data and those with a self-reported history of specific neurological illnesses (i.e., dementia, Parkinson’s disease, multiple sclerosis or stroke), or a lower-limb prosthetic leg or foot were excluded from the analyses (Appendix 1 in Supplementary materials).

### 2.2 Mobility measures

*Walking time* (in seconds) was measured with a stopwatch over a clearly marked straight-line 4-meter course. Participants were instructed to walk at their own pace. *Balance* was measured as time (in seconds) a balance position (one-legged stand, with eyes open) was held, with an upper cut-off of 60 s. Participants were positioned approximately one meter from a wall and instructed to stand on one foot for as long as possible while first lifting the dominant leg to the calf level. The test was then repeated on the other leg. In the present analysis, the longer of the two times was used. In the *chair stands* test, participants were asked to sit on a chair and fold their arms across their chest. Participants were then instructed to stand up and sit down without using their arms five times. The time (in seconds) taken to complete five chair rises was recorded.

### 2.3 Cognitive measures

Choice reaction time (in seconds), a measure of processing speed, was collected using a touch-screen computer. In this task, a row of 4 plus signs would appear in the centre of the screen. After 1 s, one of the plus signs would be replaced with a square. The participant was instructed to touch the square as quickly as possible. After 10 practice trials, the mean reaction time of correct answers, excluding timeouts, were calculated for the 52 test trials.

The Rey Auditory Verbal Learning Test (RAVLT; [9]) was used to assess immediate and delayed recall of a list of 15 words. In the CLSA, only one learning trial and one delayed trial (with a 5-minute delay) were conducted.

To assess executive function, the Mental Alternation Test (MAT; [10]) the Stroop neuropsychological screening test (Victoria version, [11]) and the phonetic and categorical fluency tests were conducted. In the MAT, participants were instructed to alternate between number and letter (i.e. 1-A, 2-B, 3-C) as quickly as possible for 30 s. The score, ranging from 0 to 51, indicates the number of correct alternations, excluding errors. The Stroop test consisted of 3 parts. First, the participant was instructed to name the colour of each circle, as fast as possible, without making mistakes. The participant was then asked to name the ink colour of each word. In the final condition, the participant is asked to name the ink of the colour words, as quickly as possible. The interference score was calculated by subtracting the time (in seconds) to complete condition 1 from the time (in seconds) taken to complete condition 3. To measure phonological fluency, participants were asked to list as many words as possible beginning with the letters “f”, “a” and “s”, in 3 60-second trials. For each trial, the score consists of the total number of permitted words given. In the present analysis, the average score from the 3 trials was used. Similarly, in the categorical fluency test a score is given based on the number of animals listed in 60 s. For further reference on the cognitive measures used, please see [12].

### 2.4 Covariates

Age, sex and education level were recorded for all participants. Education was scored on a six-point scale: (1) no qualifications, (2) completed high-school, (3) trade certificate or diploma (4) community college degree, (5) Bachelor’s degree and (6) post-graduate degree.

Additional covariates of interest were selected based on their links with mobility and/or cognition. These included depressive symptoms [13], physical activity [14], social participation [15], BMI [16] and history of arthritis [17]. Depressive symptoms were assessed using the Centre for Epidemiological Studies Depression Scale (CES-D), a clinically validated self-report questionnaire [18]. Physical activity was measured using the Physical Activity Scale for the Elderly (PASE; [20]). The PASE is a self-report questionnaire designed for older adults, and also validated in middle-aged adults, wherein participants report leisure, household, and work-related activities in the last week. Frequency of participation in social activities was measured using questions previously used in the English Longitudinal Study on Ageing [21], which addressed cultural, educational, physical, religious and familial activities participated in the last 12 months. Frequency of participation was scored on a five-point scale, ranging from (0) did not participate in a community-related activity to (4) participated in a community-related activity at least once a day. History of arthritis (rheumatoid or osteoarthritis), was collected through self-report. Finally, BMI was calculated from participant’s height and weight.

### 2.5 Statistical analyses

Descriptive summary measures are presented for all outcomes and covariates of interest. Data distributions were visually screened, and mobility and cognitive outcome values ±3 standard deviations from the mean were deemed as extreme outliers and excluded from the analysis. For balance, 13,412 (48.64%) of participants performed at ceiling. We therefore divided participants into two groups: good (held position for 60 s) and poor (held position for less than 60 s) balance. Prior to analyses, z scores were calculated for all separate tests. The z scores obtained from the processing speed task and the Stroop test were multiplied by -1 so that higher scores on all tests reflect better cognitive performance.

The primary analysis used generalised linear models to examine the relationships between mobility and cognitive outcomes, adjusting for age, sex and education level. Analyses were then repeated with depressive symptoms, physical activity, social participation, BMI and history of arthritis as additional covariates. For all analyses, standardized estimates (β), 95% confidence intervals and p-values are reported. Given that we used seven cognitive and three mobility tests, the alpha level was Bonferroni-corrected and set at *p* < 0.002.

To test the hypothesis that the association between mobility and cognition increases with age, we tested the moderating effect of age by including interaction variables in the general linear models. The interaction variable included the mobility measure and age, and these variables were centered on their means before generating higher order terms. As a secondary hypothesis, the alpha level for the interaction terms was set at *p* < 0.05. To illustrate significant interactions, the MATLAB function PlotInteraction.m was used.

Sample size varied across tests because only participants without any cognitive or mobility data were excluded. All statistical analyses were conducted using the Statistical and Machine Learning Toolbox (v10.2) for MATLAB (R2016a; MathWorks, Natwick, MA).

## 3 Results

An overview of sample characteristics is given in Table 1. The mean age was 62.9 years (SD 10.2), and 51% of the participants were female. While excluded participants did not differ in terms of education (*p* = 0.276), they were older (*p* < 0.001), more often female (*p* < 0.001), had higher BMI (*p* = 0.024) and had a higher prevalence of arthritis (*p* < 0.001) than those included in this study (Appendix 2 in Supplementary materials).

**Table 1:** Overview of sample characteristics and outcome measures.

Sample characteristics	Mean ± SD	Range
***Demographics***		
*N*	28,808	
Age (years)	62.87 ± 10.2	45–87^a^
Sex (*N*, % female)	14,683 (51%)	
Education level	4.01 ± 1.58	1–6
BMI	28.05 ± 5.43	12.9–69.65
History of arthritis (*N*, %)	9918 (34.43%)	
Depressive symptoms (CES-10)	5.19 ± 4.62	0–30
PASE score	110.63 ± 45.11	0–267.64
Social participation	3.02 ± 0.60	0–4

***Mobility measures***		
Balance (s)	39.51 ± 23.32	0–60
Chair stands (s)^b^	13.17 ± 3.35	2.12–24.66
Walking time (s)^b^	4.2 ± 0.82	1.56–7.41

***Cognitive measures***		
Choice reaction time (ms)^b^	810.1 ± 159.7	445.8–1442.8
REY Immediate recall	5.86 ± 1.84	1–11
REY Delayed recall	4.03 ± 2.1	0–10
Phonemic fluency	13.1 ± 4.27	1–35
Categorical fluency	21.46 ± 6.32	3–40
Mental alternation test	27.25 ± 7.85	1–51
Stroop score (interference)	14 ± 8.43	−83 to 122

a Although CLSA targeted men and women aged between 45 and 85 at baseline, some participants were older than 85.

b Lower values reflect better performance.

All cognitive measures were significantly associated with mobility measures after adjustment for age, sex and education (all p-values <0.001; Table 2). Standardized coefficients were small for walking speed (-0.043 to -0.106) and chair stands (-0.04 to -0.165), and small-to-moderate for balance (0.107 to 0.187). Additional adjustment for BMI, social participation, arthritis, depressive symptoms and physical activity attenuated coefficients but did not affect statistical significance (Appendix 3 in Supplementary materials).

**Table 2:** Standardized coefficients, confidence intervals (95%) and p-values for associations between measures of mobility and cognition after adjusting for age, education and sex.

	Walking time				Chair stands				Balance			
	*N*	β (SE)	95% CI	p	*N*	β (SE)	95% CI	p	*N*	β (SE)	95% CI	p
Choice reaction time	27,886	−0.106 (0.006)	−0.117, −0.094	<0.001	27,247	−0.165 (0.006)	−0.176, −0.153	<0.001	27,126	0.178 (0.013)	0.152, 0.203	<0.001
REY I	27,298	−0.064 (0.006)	−0.075, −0.052	<0.001	26,664	−0.066 (0.006)	−0.077, −0.055	<0.001	26,551	0.151 (0.013)	0.126, 0.176	<0.001
REY II	27,299	−0.043 (0.006)	−0.055, −0.032	<0.001	26,670	−0.036 (0.006)	−0.048, −0.025	<0.001	26,552	0.123 (0.013)	0.099, 0.149	<0.001
Mental alternation test	26,467	−0.096 (0.006)	−0.108, −0.084	<0.001	25,850	−0.077 (0.006)	−0.088, −0.065	<0.001	25,736	0.110 (0.013)	0.084, 0.137	<0.001
Stroop	28,333	−0.066 (0.006)	−0.077, −0.054	<0.001	27,677	−0.045 (0.006)	−0.056 −0.034	<0.001	28,734	0.124 (0.012)	0.099, 0.148	<0.001
Categorical fluency	27,613	−0.095 (0.006)	−0.110, −0.084	<0.001	26,972	−0.089 (0.006)	−0.1, −0.077	<0.001	26,846	0.181 (0.013)	0.155, 0.206	<0.001
Phonemic fluency	27,999	−0.090 (0.006)	−0.110, −0.084	<0.001	27,355	−0.095 (0.006)	−0.106, −0.083	<0.001	27,229	0.173 (0.013)	0.148, 0.199	<0.001

Beta = Standardized coefficients; SE = Standard Error.

**Fig. 1:**
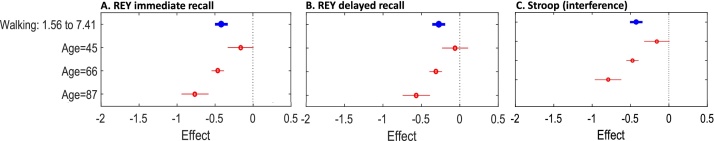
Plots illustrate the moderating effect of age on the associations between walking time and cognitive measures. Each plot shows the estimated effect when age is fixed at given values (red). (For interpretation of the references to colour in this figure legend, the reader is referred to the web version of this article.)

**Fig. 2:**
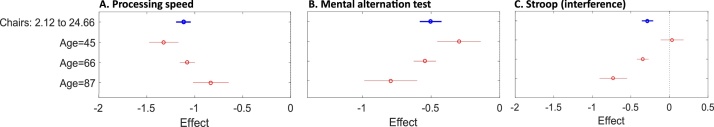
Plots illustrate the moderating effect of age on the associations between chair stands and cognitive measures. Each plot shows the estimated effect when age is fixed at given values (red). (For interpretation of the references to colour in this figure legend, the reader is referred to the web version of this article.)

**Fig. 3:**
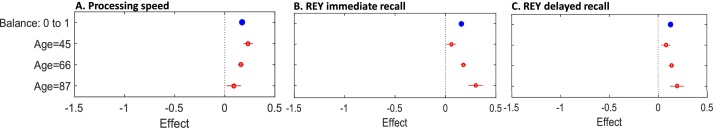
Plots illustrate the moderating effect of age on the associations between balance and cognitive measures. Each plot shows the estimated effect when age is fixed at given values (red). (For interpretation of the references to colour in this figure legend, the reader is referred to the web version of this article.)

**Fig. 4:**
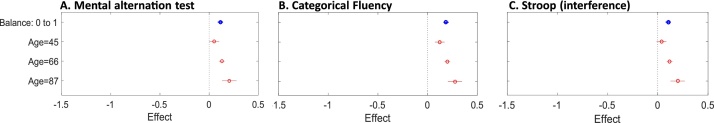
Plots illustrate the moderating effect of age on the associations between balance and cognitive measures. Each plot shows the estimated effect when age is fixed at given values (red). (For interpretation of the references to colour in this figure legend, the reader is referred to the web version of this article.)

The moderating effect of age is presented below and given in full in Appendix 4 in Supplementary materials. In Fig. 1, Fig. 2, Fig. 3, Fig. 4, each plot shows the estimated effect when age is fixed at a given value (red) and the overall estimated effect of the response, averaging out the effects of the other predictors (blue). The plotted mean effects for different age values are indicative of the role of age on the relationship between mobility and cognitive measures.

For walking time, age was a significant moderator of the association with REY immediate recall (β=-0.021, 95% CI: -0.031, -0.01, *p* < 0.001), REY delayed recall (β=-0.017, 95% CI: -0.028, -0.007, *p* = 0.002) and the Stroop test (β=-0.022, 95% CI: -0.032, -0.012, *p* < 0.001). For both REY and Stroop measures, a greater association between walking and memory was observed with increasing age (Fig. 1A–C).

For chair stands, age was a significant moderator of the association with processing speed (β = 0.018, 95% CI: 0.006, 0.029, *p* = 0.002) the mental alternation test (β=-0.018, 95% CI: -0.029, -0.007, *p* = 0.002), and the Stroop test (β=-0.027, 95% CI: -0.032, -0.012, *p<*0.001; Appendix 4 in Supplementary materials). For mental alternation and the Stroop test, an increase in age led to an increase in the association with chair rises. The opposite pattern was observed for processing speed, as measured by choice reaction time (Fig. 2A–C).

For balance, with the exception of phonetic fluency (β = 0.003, 95% CI: -0.024, 0.029, *p* = 0.842), age was a significant moderator of the relationship with all cognitive measures (Appendix 4 in Supplementary materials). The association between balance and performance on REY immediate recall (β=0.059, 95% CI: 0.033, 0.084, *p* < 0.001), REY delayed recall (β= 0.028, 95% CI: 0.002, 0.054, *p* = 0.037), mental alternation test (β= 0.037, 95% CI: 0.033, 0.084, *p* = 0.007), the Stroop test (β=0.052, 95% CI: 0.027, 0.077, *p* < 0.001) and categorical fluency (β=0.037, 95% CI: 0.011, 0.063, *p* = 0.006) increased with age. The opposite pattern was observed for processing speed, wherein a smaller effect was observed in older adults (β=-0.035, 95% CI: -0.061, -0.009, *p* = 0.009; Figs. 3,4).

## 4 Discussion

Changes in cognitive function and mobility are commonly observed in ageing populations. In a study of 28,808 community-dwelling adults we found that poor cognitive function was associated with reduced mobility, as measured by gait, balance and chair stands, even when various confounders were considered. Given the large sample size, an interpretation of standardised beta coefficients may be more informative than significance levels. It is important to stress that coefficients, albeit significant, were modest at best. The small magnitude of observed effects suggests that previous studies may have been underpowered when examining the mobility-cognition association.

It has been speculated that the mobility-cognition relationship may be selectively driven by executive function [2]. Here, we found that all cognitive measures were associated with indices of mobility. These findings are in line with a previous meta-analysis, wherein small, yet significant, pooled effect sizes for the association between mobility and executive function, processing speed and memory were reported [3]. For gait and chair stands, the largest coefficient was observed in the relationship with processing speed, while the association with memory measures tended to yield the smallest coefficients. This pattern corroborates previous reports of weak associations with memory measures [22]. Altogether, our results suggest that the association between cognition and mobility is not domain specific, but a global association, with only a suggestive predilection toward processing speed and executive function over memory measures.

Mobility is a multi-faceted concept measured in the CLSA cohort through: gait speed (locomotion), single leg-stand (balance) and chair stands (lower-extremity strength/power). Although they target slightly different aspects of mobility, observational studies have implicated gait, balance and chair stands in the loss of independence, quality of life and even mortality of older adults [23]. Here, these aspects are shown to also correlate with cognitive function. While the cross-sectional nature of our analysis prevents us from drawing conclusions regarding directionality, it could be that age-related changes in cognition affects mobility [24], that reduced mobility precedes cognitive decline [25], or that their correlation is driven by a third factor, such as physical activity [26]. Firstly, mobility relies on cognitive processes to anticipate and adapt to the moving environment while maintaining postural control and motor coordination [7]. For instance, gait and balance require the interplay of attention and executive function. Decreased executive function could, therefore, lead to decreased mobility functioning [27]. Conversely, decreased physical function, as evidenced by those with difficulties rising from chairs, balancing or walking, can promote physical inactivity, limit engagement in social and leisure activities, and increase risk of depression, all of which have detrimental effects on cognitive function [3,13,15]. Nonetheless, in the present analysis, controlling for physical activity, depressive symptoms and social activity did not remove the association between mobility and cognition.

As hypothesised, we found that the mobility-cognition association is often stronger in older adults. This increased interdependence with age may reflect age-related atrophy of shared neural structures and/or an increased reliance on cognitive processes for sensorimotor integration with age (for review, see [28]). For instance, white matter hyperintensities (WMHs) are associated with reduced cognitive function [29], slower gait and an increased risk of falls [30]. Given that WMH prevalence increases with age [29], it is plausible that increased WMH burden may account for some of the increased mobility-cognition with age. Similarly, global brain atrophy, as well as regional gray matter loss, are susceptible to age-related changes and have been shown to correlate with declines in both mobility [30] and cognitive function [31]. Finally, it is worth noting that most previous studies have looked only at older samples (aged 65+), which may explain why our standardised coefficients were smaller than those previously reported [6].

The strengths of our study include the population-based design and the many independent cognitive and mobility measures investigated. A large sample allows the incorporation of multiple confounding variables to be incorporated into the model without concerns over power loss [32]. Poor mobility is associated with a cascade of other detrimental factors (e.g. depression, social isolation, low physical activity). Many of these factors are also independently associated with cognition [[13], [14], [15]]. Analyses of the relationship between mobility and cognition should, therefore, account for these factors. To date, however, studies have often been unable to do so because they lack sample characterisation or the power to include several covariates in their models. The availability of these variables in the CLSA dataset, as well as its notable sample size, are strong advantages of this study.

Some limitations should be noted. First, analyses in large cohorts like the CLSA may yield statistically significant associations even when their explanatory power is very small [33]. For example, the association between walking and REY delayed recall was significant (p < 0.001), despite having a very small coefficient (ß = 0.04). This highlights the importance of drawing conclusions from standardized coefficients and confidence intervals over and above the p-value. Second, CLSA participants at the time of enrolment were community-dwellers aged 45-87. Recruitment excluded those living in long-term care facilities or unable to provide informed consent and this may have resulted in a healthy participant bias, particularly for the older adults. It should also be noted that our mobility measures may have lacked sensitivity. As evidenced by the high proportion of adults performing at ceiling in the balance score, a 60 s one-legged balance test may not be adequate to assess subtle balance difficulties in healthy populations. Similarly, although highly relevant in clinical populations, a 4 m self-paced walking test may not be sensitive enough to capture mobility-cognition relations in healthy adults and may be confounded by acceleration and deceleration speed at the start and end of the pathway. Accordingly, more precise (quantitative gait measures by electronic walkways), more challenging (e.g., 4 m at fast pace), or longer (e.g., 10 m at usual pace) measures may be more appropriate in research-settings. Quantitative gait analyses also offer additional gait indices, such as step-time variability and gait rhythm, which have been argued to be more sensitive to associations with cognition ([34], for review see [1]). It would, therefore, be of interest to incorporate a more comprehensive analyses of gait in future studies.

As previously mentioned, the cross-sectional design of our current findings prevents any causal inferences regarding the mobility-cognition relationship. Fortunately, CLSA participants will continue to be followed, with repeated cognitive and mobility assessments, until 2033. Driven by the baseline findings presented here, this cohort presents a unique opportunity to compare and complement cross-sectional and longitudinal findings. It will therefore be of interest to use follow-up CLSA data to address (1) whether adults with poor mobility at baseline are more likely to develop cognitive impairments later on, (2) whether adults with poor cognition at baseline are more likely to develop mobility impairments later on, and (3) whether, longitudinally, the association between the two becomes stronger with increasing age.

## 5 Conclusion

In conclusion, we found that cognitive performance was associated with indices of mobility in a large sample of community-dwelling adults. These associations remained significant even when adjusting for multiple confounders. Associations were observed across cognitive domains, and were moderated by age, suggesting that shared age-related mechanisms may underlie the relationship between mobility and cognition. While these results are informative for interventions aimed at promoting healthy ageing, the small effect sizes observed may limit the promise of mobility as a stand-alone target.

## Conflicts of interest

None.
