# Supplementary material for: Cognition and mobility show a global association in middle- and late-adulthood: Analyses from the Canadian Longitudinal Study on Aging
Source: Gait Posture. 2018 Jul;64:238–43. doi: 10.1016/j.gaitpost.2018.06.116 (PMC6052573; doi:10.1016/j.gaitpost.2018.06.116)
Supplement: Supplementary file 1 [file mmc1.docx]

**Appendices**

Appendix 1. Flow chart of participants included in the current analyses.
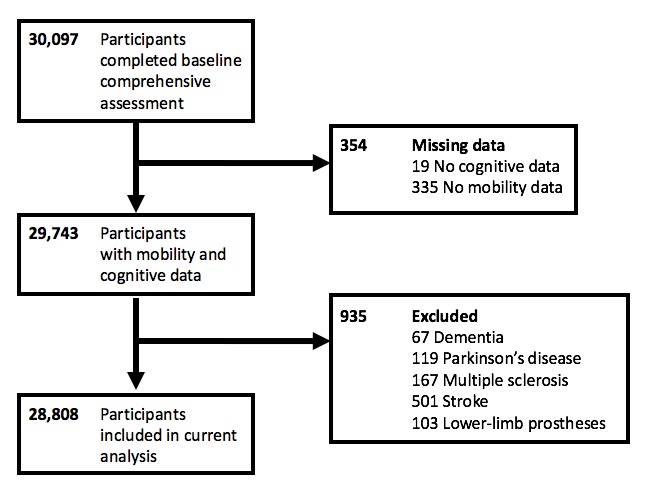


Appendix 2. Sample demographics of included (complete data) and excluded (missing data or neurological illness or lower-limb prostheses) participants.

|  | **Excluded** | **Included** | **t (df)** | ***p* - value** |
| --- | --- | --- | --- | --- |
| *N* | 1,289 | 28,808 |  |  |
| Age (years) | 67.22 ± 10.10 | 62.87 ± 10.2 | 14.99 (30,095) | < 0.001 |
| Sex (N, % female) | 637 (49.42%) | 14,683 (50.97%) | -8.66 (30,095) | < 0.001 |
| Education | 3.62 ± 1.68 | 4.01 ± 1.58 | -1.09 (30,095) | 0.276 |
| BMI | 28.41 ± 5.82 | 28.05 ± 5.43 | 2.25 (29,959) | 0.024 |
| Arthritis (N, % female) | 551 (42.75%) | 9,918 (34.43%) | 6.14 (30,095) | < 0.001 |

Values are mean ± standard deviation and *p* values are two-tailed.

Appendix 3. Standardized coefficients, confidence intervals (95%) and p-values for associations between measures of mobility and cognition after adjusting for age, education, sex, BMI, arthritis, physical activity, social participation, sleep quality and depressive symptoms.

|  | **Walking time** | | | | **Chair stands** | | | | **Balance** | | | |
| --- | --- | --- | --- | --- | --- | --- | --- | --- | --- | --- | --- | --- |
|  | *N* | β (SE) | 95% CI | p | *N* | β (SE) | 95% CI | p | *N* | β (SE) | 95% CI | p |
| **Choice reaction time** | 26,216 | -0.096 (0.006) | -0.109, -0.084 | <0.001 | 25,637 | -0.160 (0.006) | -0.171, -0.148 | <0.001 | 25,523 | 0.176 (0.014) | 0.149,  0.204 | <0.001 |
| **REY I** | 25,673 | -0.053 (0.006) | -0.065, -0.041 | <0.001 | 25,094 | -0.057 (0.006) | -0.069, -0.045 | <0.001 | 26,526 | 0.158 (0.013) | 0.132, 0.184 | <0.001 |
| **REY II** | 27,673 | -0.034 (0.006) | -0.046, -0.021 | <0.001 | 25,101 | -0.029 (0.006) | -0.041, -0.018 | <0.001 | 26,528 | 0.132 (0.013) | 0.106, 0.158 | <0.001 |
| **Mental alternation test** | 24,910 | -0.088 (0.006) | -0.101, -0.075 | <0.001 | 24,352 | -0.070 (0.006) | -0.082, -0.058 | <0.001 | 25,711 | 0.115 (0.014) | 0.088, 0.142 | <0.001 |
| **Stroop** | 26,576 | -0.049 (0.006) | -0.061, -0.037 | <0.001 | 25,988 | -0.035 (0.006) | -0.046, -0.023 | <0.001 | 27,486 | 0.089 (0.013) | 0.063,  0.115 | <0.001 |
| **Categorical fluency** | 25,950 | -0.078 (0.006) | -0.091, -0.066 | <0.001 | 25,366 | -0.077 (0.006) | -0.089, -0.065 | <0.001 | 26,820 | 0.194 (0.013) | 0.168, 0.220 | <0.001 |
| **Phonemic fluency** | 26,308 | -0.079  (0.006) | -0.091, -0.067 | <0.001 | 25,724 | -0.088 (0.006) | -0.1, -0.076 | <0.001 | 27,203 | 0.158 (0.013) | 0.132, 0.185 | <0.001 |

Appendix 4. Standardized coefficients, confidence intervals (95%), p-values and R^2^ values for all predictors in the general linear models with cognitive measures as outcomes.

|  | **Processing speed** | | | | **REY (immediate recall)** | | | | **REY (delayed recall)** | | | |
| --- | --- | --- | --- | --- | --- | --- | --- | --- | --- | --- | --- | --- |
|  | β (SE) | 95% CI | *p* | *R*^2^ | β (SE) | 95% CI | p | *R*^2^ | β (SE) | 95% CI | p | *R*^2^ |
| **Walking** |  |  |  | 0.134 |  |  |  | 0.186 |  |  |  | 0.187 |
| *Constant* | -0.002 (0.006) | -0.002, -0.021 | 0.720 |  | -0.009 (0.006) | -0.002, -0.021 | 0.105 |  | -0.007 (0.006) | -0.004, 0.018 | 0.229 |  |
| *Walking* | -0.106 (0.006) | -0.121, -0.096 | <0.001 |  | -0.059 (0.006) | -0.071, -0.047 | <0.001 |  | -0.039 (0.006) | -0.051, -0.027 | <0.001 |  |
| *Age* | -0.313 (0.006) | -0.325, -0.301 | <0.001 |  | -0.272 (0.006) | -0.284, -0.261 | <0.001 |  | -0.293 (0.006) | -0.304, -0.281 | <0.001 |  |
| *Gender* | -0.047 (0.006) | -0.058, -0.036 | <0.001 |  | 0.224 (0.006) | 0.213, 0.234 | <0.001 |  | 0.237 (0.006) | 0.226, 0.247 | <0.001 |  |
| *Education* | 0.02 (0.006) | 0.001, 0.032 | <0.001 |  | 0.185 (0.006) | 0.174, 0.196 | <0.001 |  | 0.159  (0.006) | 0.148, 0.170 | <0.001 |  |
| *Walking x age* | 0.011 (0.006) | -0.001, 0.022 | 0.053 |  | -0.021 (0.005) | -0.031, -0.01 | <0.001 |  | -0.017 (0.006) | -0.028, -0.007 | 0.002 |  |
| **Chair rises** |  |  |  | 0.147 |  |  |  | 0.184 |  |  |  | 0.186 |
| *Constant* | 0 (0.006) | -0.011, 0.011 | .999 |  | 0.006 (0.006) | -0.005, 0.017 | 0.289 |  | 0.031 (0.006) | -0.008, 0.014 | 0.586 |  |
| *Age* | -0.305 (0.006) | -0.316, -0.293 | <0.001 |  | -0.277 (0.006) | -0.029, -0.266 | <0.001 |  | -0.3  (0.006) | -0.311, -0.288 | <0.001 |  |
| *Chair rises* | -0.167 (0.006) | -0.177, -0.154 | <0.001 |  | -0.066 (0.006) | -0.077, -0.054 | <0.001 |  | -0.036 (0.006) | -0.048, -0.025 | <0.001 |  |
| *Gender* | -0.049 (0.006) | -0.060, -0.038 | <0.001 |  | 0.219 (0.006) | 0.208, 0.230 | <0.001 |  | 0.234 (0.006) | 0.223, 0.244 | <0.001 |  |
| *Education* | 0.016 (0.006) | 0.005, 0.028 | 0.006 |  | 0.185 (0.006) | 0.174, 0.196 | <0.001 |  | 0.161 (0.006) | 0.150, 0.171 | <0.001 |  |
| *Chair rises x age* | 0.018 (0.006) | 0.006, 0.029 | 0.001 |  | -0.006 (0.005) | -0.017, 0.005 | 0.282 |  | -0.004 (0.006) | -0.014, 0.007 | 0.492 |  |
| **Balance** |  |  |  | 0.128 |  |  |  | 0.184 |  |  |  | 0.186 |
| Constant | -0.039 (0.011) | -0.061, -0.018 | <0.001 |  | -0.285 (0.010) | -0.305, -0.264 | <0.001 |  | -0.293 (0.010) | -0.313, -0.272 | <0.001 |  |
| Age | -0.288 (0.009) | -0.306, -0.271 | <0.001 |  | -0.276 (0.008) | -0.292, -0.259 | <0.001 |  | -0.285 (0.008) | -0.301, -0.269 | <0.001 |  |
| Balance | 0.172 (0.013) | 0.146, 0.198 | <0.001 |  | 0.161 (0.013) | 0.135, 0.186 | <0.001 |  | 0.128 (0.012) | 0.103, 0.154 | <0.001 |  |
| Gender | -0.096 (0.011) | -0.118, -0.074 | <0.001 |  | 0.448 (0.011) | 0.427, 0.470 | <0.001 |  | 0.477 (0.011) | 0.456, 0.499 | <0.001 |  |
| Education | 0.019 (0.006) | 0.008, 0.031 | 0.001 |  | 0.183 (0.006) | 0.172, 0.194 | <0.001 |  | 0.156 (0.006) | 0.145, 0.167 | <0.001 |  |
| Balance x age | -0.035 (0.013) | -0.061, -0.009 | 0.009 |  | 0.059 (0.013) | 0.033, 0.084 | <0.001 |  | 0.028 (0.013) | 0.002, 0.054 | 0.037 |  |

|  | **Mental alternation test** | | | | **Categorical fluency** | | | | **Phonetic fluency** | | | |
| --- | --- | --- | --- | --- | --- | --- | --- | --- | --- | --- | --- | --- |
|  | β (SE) | 95% CI | *p* | *R*^2^ | β (SE) | 95% CI | p | *R*^2^ | β (SE) | 95% CI | p | *R*^2^ |
| **Walking** |  |  |  | 0.137 |  |  |  | 0.164 |  |  |  | 0.128 |
| *Constant* | 0.001 (0.006) | -0.011, 0.012 | 0.917 |  | 0.004 (0.006) | -0.008, 0.015 | 0.516 |  | 0 (0.006) | -0.011, 0.011 | 0.941 |  |
| *Age* | -0.219 (0.006) | -0.231, -0.207 | <0.001 |  | -0.266 (0.006) | -0.278, -0.255 | <0.001 |  | -0.206 (0.006) | -0.217, -0.195 | <0.001 |  |
| *Walking* | -0.094 (0.006) | -0.106, -0.081 | <0.001 |  | -0.095 (0.006) | -0.107, -0.083 | <0.001 |  | -0.085 (0.006) | -0.097, -0.074 | <0.001 |  |
| *Gender* | -0.067 (0.006) | -0.078. -0.055 | <0.001 |  | -0.023 (0.006) | -0.058, -0.036 | <0.001 |  | -0.063 (0.005) | -0.074, -0.052 | <0.001 |  |
| *Education* | 0.204 (0.006) | 0.193, 0.216 | <0.001 |  | 0.21 (0.006) | 0.199, 0.221 | <0.001 |  | 0.189 | 0.179, 0.2 | <0.001 |  |
| *Walking x age* | -0.009 (0.006) | -0.02, 0.002 | 0.103 |  | -0.003 (0.005) | -0.013, 0.008 | 0.637 |  | -0.001 (0.006) | -0.012, 0.009 | 0.808 |  |
| **Chair rises** |  |  |  | 0.132 |  |  |  | 0.161 |  |  |  | 0.117 |
| *Constant* | 0.007 (0.006) | -0.005, 0.018 | .269 |  | 0.007 (0.006) | -0.004, 0.018 | 0.231 |  | 0.009 (0.006) | -0.002, 0.021 | 0.1112 |  |
| *Age* | -0.229 (0.006) | -0.241, -0.217 | <0.001 |  | -0.274 (0.006) | -0.285, -0.262 | <0.001 |  | -0.092 (0.006) | -0.104, -0.08 | <0.001 |  |
| *Chair rises* | -0.075 (0.006) | -0.087, -0.064 | <0.001 |  | -0.088 (0.006) | -0.1, -0.077 | <0.001 |  | -0.094 (0.006) | -0.106, -0.083 | <0.001 |  |
| *Gender* | -0.074 (0.006) | -0.085, -0.062 | <0.001 |  | -0.029 (0.006) | -0.039, -0.018 | <0.001 |  | 0.099 (0.006) | 0.088, 0.11 | <0.001 |  |
| *Education* | 0.205 (0.006) | 0.193, 0.216 | 0.006 |  | 0.212 (0.006) | 0.201, 0.223 | <0.001 |  | 0.276 (0.006) | 0.264, 0.287 | <0.001 |  |
| *Chair rises x age* | -0.018 (0.006) | -0.029, -0.007 | 0.002 |  | -0.005 (0.006) | -0.016, 0.005 | 0.319 |  | -0.004 (0.006) | -0.014, 0.007 | 0.525 |  |
| **Balance** |  |  |  | 0.126 |  |  |  | 0.157 |  |  |  | 0.114 |
| Constant | 0.031 (0.011) | -0.305, -0.264 | 0.005 |  | -0.048 (0.010) | -0.069, -0.028 | <0.001 |  | -0.177 (0.017) | -0.198, -0.156 | <0.001 |  |
| Age | -0.232 (0.009) | -0.292, -0.259 | <0.001 |  | -0.263 (0.008) | -0.279, -0.246 | <0.001 |  | -0.070 (0.008) | -0.087, -0.054 | <0.001 |  |
| Balance | 0.117 (0.014) | 0.135, 0.186 | <0.001 |  | 0.187 (0.013) | 0.161, 0.212 | <0.001 |  | 0.174 (0.013) | 0.148, 0.200 | <0.001 |  |
| Gender | -0.145 (0.017) | 0.427, 0.470 | <0.001 |  | -0.050 (0.011) | -0.071, -0.028 | <0.001 |  | 0.204 (0.011) | 0.182, 0.227 | <0.001 |  |
| Education | 0.204 (0.006) | 0.172, 0.194 | <0.001 |  | 0.209 (0.006) | 0.197, 0.219 | <0.001 |  | 0.274 (0.006) | 0.263, 0.286 | <0.001 |  |
| Balance x age | 0.037 (0.014) | 0.033, 0.084 | 0.007 |  | 0.037 (0.013) | 0.011, 0.063 | 0.006 |  | 0.003 (0.014) | -0.024, 0.029 | 0.842 |  |

|  | **Stroop (interference)** | | | |
| --- | --- | --- | --- | --- |
|  | β (SE) | 95% CI | *p* | *R*^2^ |
| **Walking** |  |  |  |  |
| *Constant* | -0.011 (0.008) | -0.026, 0.004 | 0.168 | 0.177 |
| *Age* | -0.335 (0.006) | -0.346, -0.324 | <0.001 |  |
| *Walking* | -0.060 (0.006) | -0.072, -0.049 | <0.001 |  |
| *Gender* | 0.043 (0.011) | 0.022, 0.064 | <0.001 |  |
| *Education* | 0.151 (0.005) | 0.140, 0.161 | <0.001 |  |
| *Walking x age* | -0.0221 (0.005) | -0.032, -0.012 | <0.001 |  |
| **Chair rises** |  |  |  | 0.175 |
| *Constant* | -0.006 (0.008) | -0.026, 0.005 | 0.429 |  |
| *Age* | -0.347 (0.006) | -0.346, -0.324 | <0.001 |  |
| *Chair rises* | -0.043 (0.006) | -0.072, -0.049 | <0.001 |  |
| *Gender* | 0.036 (0.011) | 0.022, 0.064 | <0.001 |  |
| *Education* | 0.150 (0.005) | 0.140, 0.161 | <0.001 |  |
| *Chair rises x age* | -0.027 (0.005) | -0.032, -0.012 | <0.001 |  |
| **Balance** |  |  |  | 0.174 |
| Constant | -0.061 (0.010) | -0.081, -0.042 | <0.001 |  |
| Age | -0.345 (0.008) | 0.108, 0.157 | <0.001 |  |
| Balance | 0.132 (0.012) | -0.360, -0.329 | <0.001 |  |
| Gender | 0.038 (0.011) | 0.017, 0.059 | <0.001 |  |
| Education | 0.148 (0.005) | 0.137, 0.158 | <0.001 |  |
| Balance x age | 0.052 (0.013) | 0.027, 0.077 | <0.001 |  |
